# Nordic Crops as Alternatives to Soy—An Overview of Nutritional, Sensory, and Functional Properties

**DOI:** 10.3390/foods12132607

**Published:** 2023-07-05

**Authors:** Jaqueline Auer, Johanna Östlund, Klara Nilsson, Mathias Johansson, Anja Herneke, Maud Langton

**Affiliations:** Department of Molecular Sciences, Swedish University of Agricultural Sciences, SE-750 07 Uppsala, Sweden; johanna.ostlund@slu.se (J.Ö.); klara.nilsson@slu.se (K.N.); mathias.johansson@slu.se (M.J.); anja.herneke@slu.se (A.H.); maud.langton@slu.se (M.L.)

**Keywords:** faba bean (*Vicia faba*), pea (*Pisum sativum*), oat (*Avena sativa*), soy alternative, plant-based food, plant-based proteins

## Abstract

Soy (Glycine max) is used in a wide range of products and plays a major role in replacing animal-based products. Since the cultivation of soy is limited by cold climates, this review assessed the nutritional, sensory, and functional properties of three alternative cold-tolerant crops (faba bean (*Vicia faba*), yellow pea (*Pisum sativum*), and oat (*Avena sativa*)). Lower protein quality compared with soy and the presence of anti-nutrients are nutritional problems with all three crops, but different methods to adjust for these problems are available. Off-flavors in all pulses, including soy, and in cereals impair the sensory properties of the resulting food products, and few mitigation methods are successful. The functional properties of faba bean, pea, and oat are comparable to those of soy, which makes them usable for 3D printing, gelation, emulsification, and extrusion. Enzymatic treatment, fermentation, and fibrillation can be applied to improve the nutritional value, sensory attributes, and functional properties of all the three crops assessed, making them suitable for replacing soy in a broad range of products, although more research is needed on all attributes.

## 1. Introduction

The food industry produces 37% of total greenhouse gas emissions worldwide. The production of plant-based foods accounts for approximately 29% of these emissions, while the production of animal-based foods is responsible for more than 57% [1]. Therefore, shifting toward a more plant-based diet is one solution to reduce the environmental impact of food [1,2]. Owing to its characteristic gelling properties and its ability to form an anisotropic fiber structure [3], soy (*Glycine max*) can play a major role in supporting this dietary shift [4] and is already used in dairy [5], fish, and meat analogues [6,7]. However, in northern Europe, soy cultivation is limited by the cold climate [8]. Thus, this review explores the potential of three alternative cold-tolerant crops (faba bean (*Vicia faba*), yellow pea (*Pisum sativum*), and oat (*Avena sativa*)) to replace imported soy. Faba bean and pea are the most cultivated legumes in Sweden, with great potential for increased cultivation [9]. Both crops are primarily used as animal feed [9], but both have potential for various food applications [10,11]. Oat plays a major role in dairy replacement products [12], which is why it was also included in the review. To evaluate the potential use of the individual crops in food products, an overview of their nutritional, sensory, and functional properties was made. 

## 2. Nutritional Properties

The consumption of legumes and cereals has beneficial effects on human health [13,14], with increased consumption reported to lower the risk of heart disease [15], reduce blood cholesterol, and lower the risk of certain types of cancers [16]. While possessing these health-promoting properties, faba bean, pea, and oat have lower protein quality than soy when evaluated using the Digestible Indispensable Amino Acid Score (DIAAS) [17,18]. Table 1 provides an overview of the amino acid composition, the DIAAS and the macronutrient and micronutrient concentrations found in faba bean, pea, oat, and soy. The high DIASS score for soy (≥75) means that it is considered a high-quality protein, whereas oat, faba bean, and pea (DIAAS ≤ 75) are categorized as no-quality proteins. This can be attributed to the limited levels of cysteine and methionine in faba bean [19] and pea [20], and of lysine in oat [18]. Additionally, factors such as the folding and cross-linking of the proteins, ionic strength, and the presence of secondary molecules and antinutritional factors influence the digestibility of faba bean, pea, and oat proteins [21]. To improve the digestibility, different processing techniques can be used (see below) or different complementary protein sources can be combined to increase the DIAAS score [18].

In addition to proteins [39], micronutrients such as vitamins [40,41], minerals [36,42], β-sitosterol, and β-glucan (in oats [43]) play a major role in human nutrition. However, the presence of bioactive compounds such as tannins, phytic acid, and lectins can lower the bioavailability of certain nutrients [44,45]. For example, negative effects of phytate on the bioavailability of zinc and iron have been widely reported [46,47]. The concentrations of bioactive compounds vary between crops [48], varieties [30], growing conditions [49], and fraction of the raw material [11]. By air-classifying faba beans, Coda et al. (2015) found that anti-nutritional compounds are mostly found in the protein-rich fraction [50]. For phytate, this can be attributed to the high binding capacity between phytate and protein during seed maturation, resulting in proteins extracted from plants containing high amounts of phytic acid [51].

To improve the bioavailability of nutrients, inhibitors [52] and lectins can be fractionated during processing [53]. Lectins are inactivated during heat treatment [53]. Tannins, saponins, and phytic acid are heat-stable and can be reduced by dehulling, soaking, germination, or fermentation [19,45]. However, a recent study showed that plant-based meat substitutes available on the Swedish market, including products made from soy, pea, faba bean, and oat, lack available iron [54], despite extensive product processing. This indicates that more research efforts are needed—specifically, to reduce the phytate content in plant-based raw materials—in order to meet the dietary recommendations for iron and zinc. Aside from anti-nutrients, the compounds vicine and convicine, found in faba beans [55], can cause a severe form of hemolytic anemia in susceptible individuals [19,25]. Vicine and convicine are unstable in acidic media and degrade at higher temperatures [56], while fermentation [57] can also be used to detoxify faba beans. Despite the overall good nutritional quality of legumes and cereals, further processing is essential to improve the digestibility of proteins [21] and minerals [58] and to eliminate potentially harmful substances [56]. 

## 3. Functional Properties and Processing

The functional properties of proteins are essential for food processing and influence the organoleptic characteristics of food products. The functionalities are governed by the charge, hydrophobicity, topology, molecular weight, and structure of the proteins [59,60,61]. Plant-based proteins mainly consist of globulins [62,63,64,65] (see Table 1), which have limited solubility and a tendency to aggregate during extraction [59,66,67,68]. The functional properties of globulins differ between sources and extraction protocols, making it impossible to claim that one protein is better than another [66]. Plant-based proteins need high temperatures to undergo appreciable structural changes [59,69], further limiting their functional properties. To overcome these limitations, environmental factors such as pH, temperature, or ionic strength may need to be adjusted [61,69]. 

### 3.1. Gelation

Heat-induced protein gelation is an important step in the production of many foods. Like soy protein, faba bean and pea protein have a denaturation temperature below 95 °C [70,71,72]. Oat differs in its high denaturation temperature (approximately 110 °C) [73], which limits its gel formation in certain applications. Oat protein is reported to have poor gel properties at acidic and neutral pHs [74], although stronger gels at acidic and neutral pHs have been reported more recently [75]. The limited gelation of oat protein at certain pHs can be improved by, e.g., acylation [76]. 

Both pea and faba bean proteins have the capability to form heat-induced gels at an acidic pH [70,77]. Overall, the heat-induced gelation of pea protein appears qualitatively similar to that of soy protein [66], but pea protein forms weaker and less elastic gels when prepared under the same conditions [78]. Faba bean protein gelation has been less studied, but shows comparable behavior to soy in terms of its rheology and microstructure at different pHs and similar development of the storage modulus value during heating and cooling [23,77]. A slightly lower minimum gelling concentration has been reported for faba bean protein compared with pea and soy protein extracted using the same procedure [79].

Heating is the most common way to induce the gelation of food proteins, but gelation can also be induced by other means, such as pressure, pH shifting, and enzymatic cross-linking [80]. However, there has been little research on the gelation of oat, pea, and faba protein by methods other than heating [14,81,82,83].

### 3.2. Foaming and Emulsification

Proteins play a key role in foaming and emulsification, as they can act as a surfactant and help to stabilize foams and emulsions [84]. However, plant-based proteins typically show relatively poor foaming and emulsifying properties, so modification of the protein is necessary [84].

An alkaline pH appears to be beneficial for the foamability of faba bean protein, as it increases the surface hydrophobicity of the protein, improving air entrapment in the system [85,86]. Similar results have been reported for pea [87] and oat protein [88], although the effect of pH on foam stability varies between studies. A comparison of pea isolate and pea concentrate showed better foaming and emulsifying properties of the isolate, which was attributed to the higher purity of the protein [89]. 

Enzymatic hydrolysis increases surface hydrophobicity, and thereby foaming and emulsifying properties, at both acidic and neutral pHs. In one study, faba bean exhibited good emulsifying properties following ultrasonication and alcalase treatment, and it was able to partly replace egg yolk in low-fat mayonnaise [90]. Table 2 lists some enzymes that improve foaming and emulsion properties. Protein denaturation by high-pressure processing and heating has been shown to improve the foaming and emulsifying properties in faba bean and pea [91]. Konak et al. (2014) improved the foaming properties of oat by the selective removal of non-polar lipids using super-critical CO_2_ extraction, and they concluded that lipid-binding tryptophanins are the foam-active proteins in oat [88]. Therefore, defatting of the raw material may be necessary to produce stable foams.

### 3.3. Extrusion

During extrusion, protein molecules unfold and align in the direction of flow to form a fibrous structure supported by intermolecular bonds and aggregations [59]. Soy protein is frequently used for producing meat [7,92,93] and fish analogues [3,69], mainly using low-moisture (10–35%) extrusion (LME) [93] and high-moisture (40–80%) extrusion (HME) [94]. Products made using LME need to be rehydrated before use, which can lead to limitations in their taste and texture [7]. Therefore, only HME is discussed further here. 

Products made with HME have a high moisture content, no requirement for rehydration, and a fiber structure closer to that of meat [95]. The extrudates are generally made from soy and pea proteins, gluten [94], polysaccharides [27,96], and other non-protein ingredients that are important for the structure, flavor, and nutritional profile [7,27]. The influence of different processing parameters [97] and ingredients [98] on the product quality of soy protein-based meat alternatives is summarized by Lin et al. [99]. Pea [100,101] and faba bean [96,102] protein have the capability for producing texturized food products comparable to soy-based extrudates [3,7,103]. It has been shown that the ash, fiber, protein content, and the water-holding capacity of pea and faba bean protein affect the textural properties of the resulting meat analogues [104]. Different ratios of isolates and concentrates further affect the structure [105]. A study using HME showed that enzymatically hydrolyzed destarched oat-protein concentrate, together with pea protein, can be used to create meat analogues with a fibrillary structure [106]. Extrusion has also been used to increase the protein digestibility of pea [107] and faba bean protein [108] without causing a loss in amino acids [101] or major changes in nutritional value [19]. HME can lead to the inactivation of trypsin, chymotrypsin, and α-amylase [108] and can reduce the levels of condensed tannins and phytic acid [19]. Combined extrusion and fermentation [109] or enzymatic treatment [106] has an effect on the physicochemical, textural, and sensorial properties of extrudates [109]. Mixing different legume [110,111] and cereal-based [92,112] raw materials provides the potential to further improve their structure, nutritional value, [97] and flavor [7]. 

### 3.4. 3D Printing

Three-dimensional (3D) printing is a novel technique that allows for production of food products with personalized nutritional properties and customizable form [113]. Materials for 3D printing must be easily extrudable through the nozzle, shear-thinning, and maintain the deposited shape [114]. Challenges associated with 3D printing include ingredient mix rheology, structure accuracy, shape stability, and the effect of post-processing techniques and printing speed [115,116,117]. The emulsifying and water- and fat-binding properties of pea proteins make them a good ingredient for 3D printing [118,119]. Sridharan et al. (2021) used pea protein to stabilize rapeseed oil droplets, creating 3D-printed jammed emulsions that could retain their shape for 48 h [120]. In a subsequent study, the addition of pea protein also stabilized potato starch 3D structures by promoting cross-linking between starch granules [121]. However, if the pea protein content exceeded 2%, the protein competed with the starch for moisture and reduced the starch’s gelling abilities and paste printability [121]. In both oat and faba bean 3D-printed products, fiber is an important ingredient because of its high water-binding capacity, which improves printability and shape stability [117,122,123]. It has been shown that the undesirable color changes associated with rye and oat products can be avoided by printing at a higher pH and a lower temperature and by pre-processing the ingredients, thus reducing polyphenol oxidase activity. However, for successful 3D printing using legume and cereal protein, each ink formulation needs to be adapted individually [115].

### 3.5. Fermentation

Fermentation is used to extend shelf-life and also to impart the characteristic aromas, flavors, textures, and nutritional quality of foods [124]. Fermentation may be applied at the ingredient level, e.g., isolates, or at the product level, e.g., plant-based milk. Fermentation with lactic acid bacteria (LAB) is the main process studied in most of the available literature, while fungal and yeast fermentation are less commonly studied. Numerous LAB strains are capable of fermenting faba bean, pea, and oat, with some of these strains having been isolated from the raw material, e.g., *Pediococcus pentosaceus*, *Leuconostoc kimchi, Weissella cibaria*, and *Weissella confusa* have been isolated from faba bean [125]. *Leuconostoc plantarum* is also indigenous to plants such as legumes and grains, and it is perhaps the most studied strain for the fermentation of faba bean, pea, and oat. Moreover, commercial starters for conventional yoghurt production, containing *Streptococcus thermophilus* and *Lactobacillus delbrueckii* subsp. *Bulgaricus*, have the capability to ferment dairy analogues based on pea [126] and oat [127]. 

A considerable number of studies have observed improved nutritional quality as a result of fermentation. Increased antioxidant activity as a result of fermentation has been observed in pea flour [128,129] and oat flour [130], while degradation of condensed tannins has been found in pea flour [128] and faba bean [131] and the degradation of phytic acid in faba bean [125,132] and oat [133]. In addition, fermentation decreases the vicine and convicine levels in faba bean [131,134]. The indigestible and flatulence-causing α-galactosides found in legumes (raffinose, stachyose, and verbascose) are reported to be partly or fully degraded in fermented faba bean [125,131,135]. Oligosaccharides in oats, e.g., beta-glucan, are not altered by fermentation, suggesting the preservation of pre-biotic properties [133]. 

The proteolytic effect of LAB has been verified by the documented release of free amino acids in pea [128] and faba bean [131]. The latter study demonstrated improved in vitro protein digestibility and increased concentrations of the amino acids cysteine and methionine. As a consequence of altered structure and composition, the functional properties of the protein may change, resulting, e.g., in increased protein solubility, foaming capacity, and stability in faba bean flour [135]. In contrast, protein solubility, foaming, and emulsifying properties are hampered in fermented pea flour [136] and pea protein isolate [137]. Similar findings have been reported for pea proteins, with the exception that emulsification is not affected by fermentation. Furthermore, fermentation has been proven to be a successful method for the removal of off-flavors in soy, with recent findings suggesting similar effects from the fermentation of pea protein [138] and a pea-based milk analogue [126]. 

### 3.6. Enzymatic Treatment

As in fermentation, enzymes are used to catalyze specific chemical reactions and thereby improve the taste and texture of food [139,140]. An overview of the most commonly used enzymes and their effect on different proteins and functional properties are shown in Table 2. Proteolytic enzymes such as pepsin, papain, trypsin, and alcalase are used to hydrolyze peptide links between amino acids [59,141]. This leads to changes in molecular weight and tertiary structure that can increase the surface hydrophobicity, solubility, emulsifying, and foaming properties of pea [59,142,143], faba bean [144,145,146], and oat protein [24,26,147]. Trypsin hydrolyzation has been shown to increase the foaming capacity of oat protein [148,149], while trypsin and papain have been used to improve the emulsifying and foaming properties of pea protein [150]. While improving the functional properties, the size reduction of proteins can promote a bitter taste [142,151]. The bitterness of the hydrolysates can be attributed to the occurrence of hydrophobic amino acids and is correlated with the degree of hydrolysis [59,142,152]. To reduce the bitterness in pea protein hydrolysates, sec-butanol and β-cyclodextrin treatment has been used [153]. Fermentation followed by enzymatic treatment has the ability to reduce off-flavors, with a simultaneous increase in functional properties [152]. Hydrolysis and fermentation of oat can also increase its soluble and insoluble phenolic content and thus produce functional food ingredients [154]. When producing milk analogues from oat, enzymatic hydrolysis is used to avoid the gelatinization of starch during the heat treatment [5]. Non-proteolytic enzymes such as transglutaminases can be used to improve the functional properties of food proteins by cross-linking glutamine and lysine residues [52,59]. Transglutaminases can thereby influence the viscosity, emulsification [155,156], gelation [157,158], and foaming properties and increase the hydrophobicity of food proteins [72,159]. All enzymatic reactions depend strongly on the polypeptide composition, protein conformation, and reaction conditions, and therefore require individual adaptation to a specific raw material [160,161,162,163]. Apart from improving the functional properties, enzymes can also be used to improve the nutritional quality of the resulting food. By reducing the amount of phytate, especially the amount of inositol hexaphosphate, through the addition of phytase, the digestibility of proteins [164] and minerals can be improved [109,165]. Phytase is currently used only in animal feed products and has not been approved for food products [166], but the use of phytase can become a promising future tool for improving the nutritional quality of legumes and cereals.

### 3.7. Protein Nanofibrillation

Protein nanofibrils (PNFs) from plant-based protein sources have great potential to create new textured foodstuffs. PNFs are ribbon-like, β-sheet-rich aggregates linked together with hydrogen bonds in a cross-β-sheet structure [167]. Most food-related PNFs are formed at pH 2–3 and 80–90 °C, enabling the protein to unfold and hydrolyze the structure into smaller peptides [168]. Nucleation and elongation of the ribbon-like fibril are further promoted by the peptides formed; mature fibrils usually have a high aspect ratio only some nm thick and several µm long. The hierarchical structure of the PNFs gives them high stiffness and great stability against harsh environments, which could be used to improve the functional properties of food applications [167,169,170]. PNFs have been found to stabilize colloid dispersions such as foams and emulsions [171,172,173,174,175,176], gels [177], and hydrogel fibers [178], and to be efficient carriers of nutrients such as iron particles [179,180]. 

In recent years, researchers have focused on the formation and functional properties of plant-based PNFs [159]. Protein isolates from soybean, faba bean, pea, and oat are reported to form PNFs under acidic conditions and at high temperatures. However, the PNFs formed have some differences in morphology, e.g., heat-incubated acidic protein isolates from soybean and faba bean can form both straight [176,180,181] and curved [181,182] PNFs, whereas pea protein has been reported to form curved PNFs [183] and oat protein mainly forms PNFs with straight morphology [182,184]. Curved PNF samples have been reported to have higher viscosity compared with straight PNFs due to their more flexible structure, making them promising candidates for stabilizing colloid systems [185,186].

A few in vitro [187,188] and in vivo [179] studies that have been conducted reviewing the digestibility and toxicity of PNFs concluded that there is no major negative impact of consuming PNFs. Food-related PNFs have structural similarities to some disease-related amyloid fibrils. This has led to a concern that PNFs made from food protein could have a seeding effect on the disease-related amyloids. However, a newly published study by Rhaman et al. (2023) showed that PNFs from proteins such as faba bean and oat do not accelerate the aggregation of amyloid-β (Aβ), a peptide related to the development of Alzheimer’s disease [189]. This result indicates that plant-based PNFs have a future as texture enhancers in future food applications.

## 4. Sensory Properties

Most of the research on the sensory properties of legumes and oat as components in plant-based foods has focused on the identification and mitigation of off-flavors. Faba bean and pea are frequently described as “beany”, “grassy”, “astringent”, and “bitter” in taste [196,197], while the latter term is also associated with oat [198]. However, the meaning of these descriptions is not consistent throughout the literature [197]. The complexity of off-flavors is due to the large number of compounds that contribute to flavor. In addition, consumer perception varies greatly depending on the food matrix and the presence of other compounds with sometimes very low taste detection thresholds (1 ppm), where minor changes can have a large impact on perception [199]. An example of a volatile compound that is responsible for the beany and grassy flavor at low levels is hexanal, a breakdown product of unsaturated fatty acids during lipid oxidation [200,201]. 

Off-flavors in oat, faba bean, and pea arise from the breakdown of lipids and protein and/or the presence of non-volatiles such as phenolic compounds, tannins, alkaloids, and saponins [196]. Lipid oxidation is reported to be the main cause of off-flavors [199], but the molecular pathway, concentration, and type of volatile depend on the crop variety, harvesting and storage conditions, and food processing [202]. Enzymatic lipid oxidation dominates in legumes, owing to the high activity of lipases and lipoxygenases [200], whereas autoxidation is more prominent in oat [203]. 

The amount of key off-flavors present is strongly associated with the activity of lipoxygenases, rather than the presence of lipids [204], and thus faba bean and pea may develop more lipid-derived off-flavors than oat despite their much lower lipid content. A comparison of soybean, pea, and faba bean showed 3-fold higher lipoxygenase activity in soy compared with faba bean (2-fold higher activity when protein extracts were compared), while pea had slightly higher activity than faba bean [205]. Another group of low-threshold volatiles are the pyrazines, which occur naturally in peas but can also originate from Maillard reaction and which give rise to several off-flavor derivatives [206].

Although the inherent characteristic flavors of pulses are accepted by consumers to some extent, the majority of published articles focus on the identification and mitigation of these off-flavors to improve sensory properties [207]. The milling of pulses into flour is a process step in which volatiles are formed, but their abundance is influenced by the settings of the mill [208]. Karolkowski et al. (2023) compared fractions of air-classified faba bean flour and found higher concentrations of volatiles and lipids in the protein fraction compared with the starch fraction [209]. Similarly, Murat et al. (2013) found more volatiles in the pea protein isolate compared with pea flour [210]. Many of the identified volatiles have been further characterized and classified into odor categories. However, knowledge about consumer perception of these odors in pulse-based foods is scarce. Studies have shown that faba bean flour can partially substitute wheat flour in bread and pasta without affecting the sensory profile negatively [211,212,213]. In addition, faba bean has been included in meat substitutes [102] and mayonnaise [214] without negatively altering their sensory properties. Badjona et al. (2023) discuss consumer acceptability of food formulations using faba beans in more detail [215]. 

Thermal processing to inactivate enzymes is reported to be a successful method for minimizing off-flavors in faba beans and peas [216,217,218]. Other methods to mitigate or mask off-flavors include soaking and enzymatic treatment to remove bitter compounds, pH modulation, fermentation, use of flavor additives, and solvent extraction. Table 2 presents some examples of enzymes that can improve sensory characteristics. Detailed descriptions of different methods for improving off-flavors can be found in the comprehensive review by Wang et al. [199]. 

## 5. Conclusions

Soy protein has unique nutritional, sensory, and functional properties that make it versatile. Pea, faba bean, and oat have nutritional and functional properties that can compete with soy, although the overall protein quality is lower. Combining different protein sources can balance the amino acid composition, but the presence of antinutrients can reduce the overall digestibility of macro- and micro-nutrients. Antinutrient concentrations can be reduced during processing, but recent studies have shown that even highly processed plant-based foods are limited in terms of their iron and zinc availability [54]. Therefore, more research is needed on processing methods and the identification of materials low in phytic acid. Furthermore, the extraction and classification method can have a major effect on the functional, nutritional and sensory properties [23] and represent key processing steps in obtaining products with a desirable taste and texture, but also with adequate nutritional properties. Moreover, vicine and convicine should be treated as allergens, as the consumption of these compounds can have severe consequences for susceptible individuals.

The removal of off-flavors remains a major challenge when faba bean, pea, and oat are processed into foods. This review covered the origin and ways to mitigate off-flavors, but more research is needed to optimize treatments for specific crops. Furthermore, more studies that correlate the odor profile with consumer perception are needed. 

Gel formation and gel properties depend largely on the extraction process and conditions used during gelation. Further research is needed to identify the underlying reasons and mechanisms for these differences. In general, heat-induced soy protein gels tend to be stronger than those prepared from faba bean and pea. However, the overall gel formation and behavior in response to changes in pH and salt appear to be similar for pea, faba bean, and soy protein. Future research should also explore the gelation induced by other means, such as pressure, pH shifting, and enzymatic cross-linking. More studies on foamability and emulsification at different pHs are needed since existing studies show inconsistent results. Enzymatic treatments can successfully improve the sensory and functional properties of faba bean, pea, and oat and also have the potential to improve their nutritional value. There is great potential to use protein, starch, and fiber extracted from faba bean, pea, and oat to create texturized food products based on techniques such as 3D printing, extrusion, or reconstruction of the protein into PNFs. Although extrusion is the most commonly used method today for creating meat analogues, there are limitations in the length of fibers that can be produced. Making meat-like fibers in a bottom-up approach using 3D printing or by PNFs could be a future complement to extruded products to create plant-based products with longer meat-like fibers and a controlled structure. However, there is still much research to be done, such as scalability and proper safety studies on the digestibility and bioavailability of PNFs. 

Decades of research made soy the commodity it is today. Further research on the nutritional, sensory, and functional properties of the cold-climate crops faba bean, pea, and oat as potential texturizing ingredients to replace soy could make them the commodities of the future. 

## Figures and Tables

**Table 1:** Concentrations of macronutrients and micronutrients in soy, pea, faba bean, and oat protein and their DIAAS score, where DIAAS ≥ 100 indicates an excellent protein source meeting all physiological requirements [17,18].

	Soy	Pea	Faba Bean	Oat		Sources
Carbohydrates (%) ^a^	20–30	28–65	55–71	69–76		[3,4,10,11,22,23,24,25,26,27,28,29]
Protein (%) ^a^	36–40	23–31	23–35	12–20		
Globulins (%)	90	49–70	60–78	70–80		
Prolamins (%)	NA	4–5	3–8	4–14		
Albumins (%)	NA	15–25	18–22	1–12		
Glutelins (%)	NA	11	10–18	<10		
Fiber (%) ^a^	5–12	22–27	4–17	5–10		
Fat (%) ^a^	18–20	1–2	0.7–3.2	5–18		
Saturated fatty acids (%)	6	18–25	0.4	19–22		
Monounsaturated fatty acids (%)	21	22–33	0.6	36–40		
Polyunsaturated fatty acids (%)	73	42–61	1.3	40–43		
Ash (%) ^a^	5–6	2–4	2.6–4.4	1–2		
Amino acids (mg/g protein) Essential amino acids					Recommended intake ^d^, mg/g	
Histidine	16.5	20.0	29.2 ^b^	14.1	15	[24,30]
Isoleucine	20.9	28.8	24.5 ^b^	20.3	30	
Leucine	55.0	71.3	57.8 ^b^	59.4	59	
Lysine	37.4	58.8	52.5 ^b^	20.3	45	
Methionine	3.3	3.8	NA	1.6		
Phenylalanine	35.2	46.3	30.7 ^b^	42.2	30 *	
Threonine	25.3	31.3	31.3 ^b^	23.4	23	
Valine	24.2	33.8	37.1 ^b^	31.3	39	
Non-essential amino acids						
Arginine	52.8	73.8	93.2 ^b^	48.4		
Alanine	30.8	40.0	52.6 ^a^	34.4		
Cysteine	2.2	2.5	10.01 ^b^	6.3		
Glutamic acid	136.3	161.3	144.7 ^a^	171.9		
Glycine	29.7	35.0	34.1 ^a^	26.6		
Proline	36.3	38.8	37.7 ^a^	39.1		
Serine	37.4	45.0	44.3 ^a^	34.4		
Tyrosine	24.2	32.6	NA	23.4		
DIAAS ^c^	91–92	66–70	55	44–57		[17,18]
Vitamins ^a^						[19,31,32,33,34]
Vitamin B1 (mg)	1.0	0.7	0.55	1.1		
Vitamin B2 (mg)	0.46	0.27	0.29	0.18		
Vitamin B3 (mg)	3.6	NA	2.8	0.9		
Vitamin B6 (mg)	1.1	0.12	0.37	0.15		
Vitamin B7 (µg)	30	NA	NA	38		
Vitamin B9 (µg)	250	274	423	37		
Vitamin E (mg)	29.5	5.11	0.08	3.3		
Elements ^a^						[11,33,34,35,36]
Calcium (ppm)	2680	850	1030	100		
Magnesium (ppm)	1820	1450	1500	1600		
Potassium (ppm)	12,690	10,000	13,300	5100		
Phosphorus (ppm)	6177	5500	6560	4100		
Zinc (ppm)	40	43	48.5	40.8		
Copper (ppm)	14	7	16.6	8.6		
Iron (ppm)	91	60	26.5	94.1		
Manganese (ppm)	NA	NA	14.4	40.3		

^a^ per 100 g of the major bean/grain. ^b^ average of different cultivars. ^c^ FAO FN Paper 92 2011, ages 0.5–3 years, AA ref standard (mg/g protein) [37]. ^d^ mean nitrogen requirement of 105 mg/kg per day (0.66 g protein/kg per day) [38]. * value corresponds to phenylalanine + tyrosine [38].

**Table 2:** Summary of most commonly used enzymes and their effects on the structure and functional properties of soy, pea, faba bean, and oat protein.

Enzyme	Mechanism and Impact on the Structure	Soy	Pea	Faba Bean	Oat
		**Improved functional properties**			
**α-Chymotrypsin^α-C^** **Alcalase^Al^** **Flavorzyme^Fl^** **Papain^Pa^** **Pepsin^Pe^** **Trypsin^Tr^** **Transglutaminase^Tr^** **Tyrosinase^Ty^** **Glutaminase^Pg^**	Hydrolysis of peptide bonds on the C-terminal side of tryptophan, tyrosine, phenylalanine, and leucine Nonspecific serine-type protease used to produce protein hydrolysates with better nutritional or functional properties Selective release of hydrophobic amino acid residues, producing a debittering effect Cleaves the peptide bonds of leucine and glycine; also hydrolyzes esters and amides Cleaves peptide bonds at the amino-terminal side of tyrosine, phenylalanine, and tryptophan Cleaves peptide bonds between the carboxyl group of arginine or lysine and the amino group of the adjacent amino acid Cross-linking between glutamine and lysine residues Oxidase protein-bound tyrosine residues which further react with tyrosines, lysines, or cysteines Catalyzes the deamidation of glutamine residues or polypeptides into glutamic acid	↑solubility (pH 4; pH 7) ^Al, Pa, Pe, Fl^ ↑foaming properties (DH 2.5–10%) ^Al + Tg^ ↑foaming activity (pH 2; pH 6–8) ^Al, Pa, Pe, Fl^ ↑sensory characteristics (improves with DH; 120 min) ^Fl^ ↑water-binding capacity ^Pa^ ↑oil-binding capacity (DH 2.5–10%) ^Al, Pa, Pe, Fl^ ↑emulsifying properties (DH 2.5–5%) ^Al + Tg^ ↓ allergen fractions ^Al, Pa, Pe, Fl^ ↑masking bitter peptides ^Al + Tg^	↑solubility (DH 3.7, 6.8%; pH 4.6–7) ^Pa, Tr^ ↑foaming capacity (DH 3.7, 6.8%) ^Pa, Pe, Tr^ ↑foaming stability (pH 7; pH 10) ^Pa, Pe, Tr^ ↑sensory characteristics (improves with DH) ^Pa, Tr, α-C^ ↑water-holding capacity (DH 2.3–11.3%) ^Pa, Pe, Tr, Tg^ ↑oil-holding capacity (DH 2.3–11.3%) ^Pa, Pe, Tr^ ↑emulsifying capacity (hydrolysis 15–120 min) ^Tr, α-C^ ↓ allergen fractions ^Pa, Tr^ ↑gelling properties (pH 6.5) ^Tg^	↑solubility (time of hydrolysis, 15–60 min; pH 5; pH 7; 10–10,000 nkat/g) ^Al, Pe, Tr, Fl, Tg, Ty^ ↑foaming properties (time of hydrolysis, 15–60 min; pH5; pH 7) ^Ty, Tg, Pe, Tr, Fl^ ↑oil-holding capacity (time of hydrolysis, 15–60 min; pH7) ^Pe^ ↑emulsion stability (time of hydrolysis, 15 min; pH7) ^Tr, Fl^ ↑colloidal stability (DH 9, 16%) ^Ty, Tg^	↑ solubility (pH 4; pH 7; 10–10,000 nkat/g) ^Al, Tr, Tg, Pg^ ↑foaming properties (pH4; pH 7; 10,000 nkat/g) ^Al, Tr, Tg^ ↑emulsifying properties (pH 4.5; pH7; pH 10.5) ^Pg, Al, Tr^ ↑colloidal stability (100–10,000 nkat/g) ^Tg^ ↑gelling properties (pH4; pH7) ^Al, Tr^
		**Reduced functional properties**			
		↓ water-binding capacity (DH 2.5–10%) ^Al, Pe, Fl^ ↑bitterness (hydrolysis 10–120 min) ^Al, Pa, Pe^ ↓ foaming stability (pH 2; pH 6–8) ^Al, Pa, Pe, Fl^ ↓ foaming density (pH 6–8) ^Al, Pa, Pe, Fl^ ↓ emulsifying properties (DH 10) ^Al + Tg^	↓ solubility (DH 2.3–11.3%; pH 4–10) ^Pa, Pe, Tr, α-C^ ↓ foaming capacity (DH 2.3–11.3%; pH 4–10) ^Tr, Pa, Pe^ ↓ emulsifying capacity (DH 3.7%) ^Pa, Tr^ ↓ emulsifying stability (pH 4; pH 7; pH 10) ^Pa, Pe, Tr^	↓ foaming properties (pH 7; 10–10,000 nkat/g) ^Ty, Tg^ ↓ emulsion stability (time of hydrolysis, 15–60 min) ^Pe, Tr^	↓ solubility (pH 7; 10–10,000 nkat/g) ^Ty^ ↓ foaming properties (pH 7; 10 nkat/g) ^Ty^
**Reference**	[160,190,191]	[141,192,193]	[152,155,157,158,194]	[72,144,195]	[24,72,149,191]

↑ improved/increased specific functional properties; ↓ reduced/decreased specific functional properties; DH: degree of hydrolysis. Different superscript letters refer to the individual enzymes where a certain effect was observed.

## Data Availability

The data used to support the findings of this study can be made available by the corresponding author upon request.
